# Kinetics of the viral cycle influence pharmacodynamics of antiretroviral therapy

**DOI:** 10.1186/1745-6150-6-42

**Published:** 2011-09-12

**Authors:** Ahmad R Sedaghat, Claus O Wilke

**Affiliations:** 1 Department of Otolaryngology, Massachusetts Eye and Ear Infirmary, Harvard Medical School, Boston, MA, USA; 2 Section of Integrative Biology, Center for Computational Biology and Bioinformatics, and Institute for Cell and Molecular Biology, The University of Texas at Austin, Austin, TX 78712, USA

**Keywords:** HIV, viral dynamics, HAART, antiretroviral therapy, viral life cycle, pharmacodynamics, IC50

## Abstract

**Background:**

More and more antiretroviral therapies are being developed for treatment of HIV infection. The *in-vivo *efficacy of these drugs is commonly predicted based on *in-vitro *measures of antiviral effect. One primary *in-vitro *measure is the IC50, the amount of drug required for 50% inhibition of viral replication. We have previously shown that HIV life-cycle kinetics impact clinically observed HIV viral dynamics. Here we present a mathematical model of how they affect the pharmacodynamics of antiretroviral drugs.

**Results:**

We find that experimentally measured antiretroviral IC50s are determined by three factors: (i) intrinsic drug properties (e.g. drug-target binding), (ii) kinetics of the HIV life cycle, and (iii) kinetics of drug-inhibited infected cells. Our model predicts that the IC50 is a declining function of the duration of the drug-susceptible stage in the host cell. We combine our model with known viral life-cycle kinetics to derive a measure of intrinsic properties, reflecting drug action, for known antiretroviral drugs from previously measured IC50s. We show that this measure of intrinsic drug property correlates very well with *in vitro*-measured antiviral activity, whereas experimentally measured IC50 does not.

**Conclusions:**

Our results have implications for understanding pharmacodynamics of and improving activity of antiretroviral drugs. Our findings predict that drug activity can be improved through co-administration of synergistic drugs that delay the viral life cycle but are not inhibitory by themselves. Moreover, our results may easily extend to treatment of other pathogens.

This article was reviewed by Dr. Ruy Ribeiro, Dr. Ha Youn Lee, Dr. Alan Perelson and Dr. Christoph Adami.

## Background

In the last twenty-five years, dramatic improvements have been made in highly active antiretroviral therapy (HAART) against the human immunodeficiency virus (HIV) [1-3]. As more effective and novel classes of antiretroviral therapies are developed, it becomes increasingly important to understand the mechanism of action for these medications.

Two pharmacodynamic properties primarily determine the activity of a drug: the IC50, i.e., the concentration of drug required for 50% inhibition, and the sigmoidicity of the concentration-response curve, as determined by a Hill coefficient. Using a novel *in vitro *drug assay [4], a recent study reported pharmacodynamic parameters of IC50 and Hill coefficient for over 25 antiretroviral drugs within the nucleoside-analogue reverse transcriptase inhibitors (nRTIs), non-nucleoside-analogue reverse transcriptase inhibitors (nnRTIs), protease inhibitors (PIs), integrase inhibitors (INIs), and fusion inhibitors (FIs) [5]. This work revealed drug-class-specific pharmacodynamic properties. Certain drug classes may be more efficacious than others by virtue of greater sigmoidicity of the concentration-response curve--with some protease inhibitors alone exhibiting greater than nine logs suppression of viral replication [5] in CD4+ T cells. While there is now a preponderance of data describing the activity of different antiretroviral drugs, the impact of viral life cycle kinetics on antiviral pharmacodynamics remains unclear.

We and others have previously observed that the kinetics of the viral life cycle may quantitatively impact viral dynamics in response to treatment [6-8,8]. Here we explore how the kinetics of the viral life cycle may influence the pharmacodynamics of antiretroviral drugs, using a general and simple mechanistic mathematical model of HIV antiretroviral pharmacodynamics. We find that classical measures of potency such as the IC50 may be functions of viral life-cycle kinetics. We explore how pharmacodynamics would be expected to vary between different virus-producing cell types and how viral life-cycle kinetics may be manipulated to improve drug efficacy. Finally, we use our model to extract information about properties of known antiretroviral drugs. IC50 tends to be a poor reflection of antiviral activity when comparing antiviral activity across drug classes such because the IC50 of antiretroviral drugs correlates poorly with suppression of viral replication at physiologic drug concentrations [5]. We use our model to eliminate the confounding effects of viral life-cycle kinetics from IC50, deriving a measure of intrinsic drug activity that correlates strongly with antiviral activity as measured by the instantaneous inhibitory potential in a single round cell-culture assay system. Our findings offer novel mechanistic insight into the efficacy of antiviral drugs and suggest novel approaches for maximizing the efficacy of HAART.

## Methods

### A mathematical model of antiretroviral drug action

The pharmacologic effect of an antiretroviral drug on viral replication in *in-vitro *assays has been described with non-linear sigmoidal models, such as the Median-Effect model by Chou and Talalay [9]. This model states that *f*_unaffected_, the fraction of HIV-infected cells that are uninhibited and proceed through the viral life cycle, is given by

(1) $$f_{\text{unaffected}}=\frac{1}{1+{\left(\frac{\left[c\right]}{IC_{50}}\right)}^{m}},$$

where [*c*] is the concentration of drug, IC50 is the concentration of drug that produces a 50% reduction in maximal viral replication, and *m *is a Hill coefficient that determines the sigmoidicity of the concentration-response curve and may reflect cooperativity at the level of molecular binding. While such non-linear sigmoidal models can offer insights into antiretroviral activity, e.g. through fitting of the IC50 or the Hill coefficient *m *to concentration-response data, they cannot give mechanistic insight because HIV viral dynamics do not meet important assumptions underlying these models [10]. In any situation, if biology obviously violates model assumptions, then the model cannot be trusted to generate accurate predictions, even if it fits available data. Specifically, HIV viral dynamics do not meet the assumptions of (i) free diffusion of drug and the substrate (HIV enzymes are spatially restricted to the virion or infected cell and therefore an antiretroviral drug molecule may only act on HIV enzymes in the same virion or infected cell) and (ii) enzyme recycling (once a single reverse transcriptase molecule acts in an infected cell, it cannot go to another infected cell to act).

**Figure 1:**
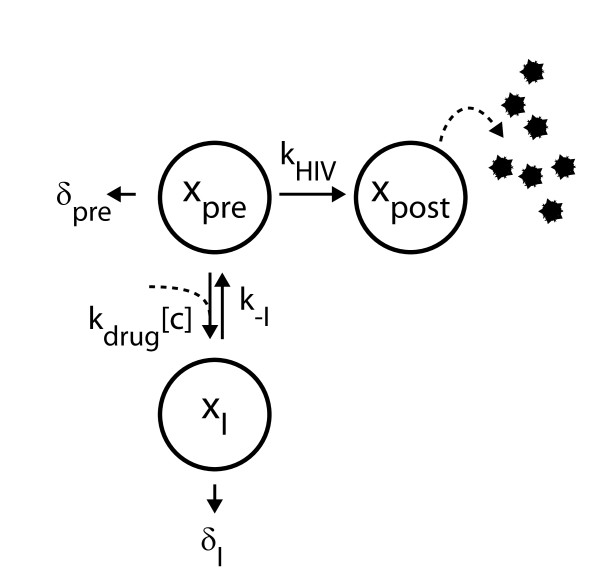
**Schematic of the model**. An HIV-infected cell type (*x*_pre_) that is susceptible to an antiretroviral targeting the step in the viral life cycle that proceeds at rate *k*_HIV _after which the infected cell is no longer susceptible to the antiretroviral drug (*x*_post_). The drug-susceptible cell can be inhibited at rate *k*_I _to become an inhibited cell (*x*_I_), which can progress no further in the HIV viral life cycle. This inhibition may be reversed at a rate *k*_-I_. Cell types *x*_pre _and *x*_I _decay (e.g. through death of the cell or decay of the offending HIV virion) at rates *δ*_pre _and *δ*_I_, respectively.

To study how the stage at which a drug acts may affect pharmacodynamics we present a general and simple mechanistic mathematical model of HIV kinetics that can be applied to any class of antiretroviral drug or any virus-producing cell type in the setting of pharmacologic inhibition (Figure 1). This model is constructed to reflect the in-vitro experiments that are used to quantify antiretroviral pharmacodynamic parameters such as IC50. Our model is defined by three variables corresponding to different stages of the viral life cycle within infected cells (e.g. reverse transcription, integration, maturation): *x*_pre _represents the stage that is susceptible to the drug effects; *x*_post _represents the stages not susceptible to the drug; these stages will produce virus; *x*_I _represents stages that are being actively inhibited by the drug. For example, if applying this model to studying reverse transcriptase inhibitors, *x*_pre _would represent infected cells undergoing reverse transcription, which would be susceptible to reverse transcriptase inhibitors, *x*_post _would represent infected cells that have progressed beyond reverse transcription, and *x*_I _would represent infected cells that are being actively inhibited by a reverse transcriptase inhibitor.

This model is defined by the system of ordinary differential equations:

(2) $$\frac{dx_{\text{pre}}}{dt}=k_{\text{ - I}}x_{\text{I}}-\left(k_{\text{I}}+\delta_{\text{pre}}+k_{\text{HIV}}\right)x_{\text{pre}},$$

(3) $$\frac{dx_{\text{post}}}{dt}=k_{\text{HIV}}x_{\text{pre}},$$

(4) $$\frac{dx_{\text{I}}}{dt}=k_{\text{I}}x_{\text{pre}}-\left(k_{\text{ - I}}+\delta_{\text{I}}\right)x_{\text{I}}.$$

Here, *k*_HIV _is the rate at which the drug-susceptible state transitions to a non-susceptible state. In our example of reverse-transcriptase inhibitors, *k*_HIV _would be the rate of reverse transcription. *k*_I _is the rate at which viral replication in susceptible cells (*x*_pre_) is inhibited; *k*_-I _is the rate at which the inhibition of susceptible stages decays. The parameters *δ*_pre _and *δ*_I _represent the decay rates for susceptible and inhibited cell stages. Cell death is ignored in the *x*_post _state because it is unlikely to play a major role under the experimental protocol used to generate published data [5]. Following classic enzyme kinetics and the law of mass action, the rate *k*_I _is a function of the drug's interaction with the target cell. The interaction of a drug with the target cell includes uptake, intracellular transport, drug binding to its target (designated *k*_drug_), and the drug concentration [*c*], and is given by *k*_I _= *k*_drug _[*c*]*^m^*. The parameter *m *determines the sigmoidicity of the concentration-response curve. As yet, the mechanism underlying the value of *m *is unknown and its value is empirically determined through fitting of experimental data to a concentration-response curve. We will consider *k*_I _to be a constant because *k*_drug _is a constant and drug concentration is approximately constant in short term drug assays where pharmacodynamic experiments are performed.

For model simulations and calculations, we made parameter choices based on published results for the kinetics of HIV infected CD4+ T cells [11] and the kinetics of the different drug classes in CD4+ T cells [12-17].

## Results

### Intrinsic and extrinsic factors affect pharmacodynamics

A concentration-response curve for any given enzyme inhibitor (e.g. a drug) is the plot of enzyme activity in the presence of inhibitor against inhibitor concentration. In the case of antiretroviral drugs, enzyme activity is indirectly measured in assays as the fraction of infected target cells that proceed through the HIV viral life cycle compared to the number of such cells in the absence of the drug. In our model, we can calculate the fraction *f*_unaffected _of uninhibited viral replication given a specific drug concentration by taking the asymptotic ratio of *x*_post _in the presence and the absence of the drug. Thus, we have $f_{\text{unaffected}}={\left[x_{\text{post}}\left(\infty \right)\right]}_{k_{\text{I}}\neq 0}∕{\left[x_{\text{post}}\left(\infty \right)\right]}_{k_{\text{I}}=0}$. For our model, we obtain:

(5) $$f_{\text{unaffected}}=\frac{1}{1+\frac{\delta_{\text{I}}k_{\text{drug}}{\left[c\right]}^{m}}{\left(\delta_{\text{pre}}+k_{\text{HIV}}\right)\left(k_{\text{ - I}}+\delta_{\text{I}}\right)}}.$$

Equation 5 is in the same form as the Median-Effect model (equation 1) where the Hill coefficient is similarly equal to *m *(and may reflect the order of the reaction or molecular level cooperativity). By definition, the IC50 corresponds to the drug concentration [*c*]at which *f*_unaffected _= 1/2. We find

(6) IC50=[(δpre+kHIV)(k-I+δI)δIkdrug]1m.

Thus, our model links fundamental constants that describe how a drug acts to the experimentally observable IC50. Equation 6 suggests that three factors play a role in determining the IC50 of a drug: intrinsic properties of the drug, the kinetics of the viral life cycle, and the kinetics of inhibited infected cells. The intrinsic properties of a drug (*k*_drug_) include how well it is taken up, complexes with, and inhibits its target, and how easily the reverse process takes place (*k*_*-I*_). When a drug accesses its target better (larger *k*_drug_), the IC50 is lower. The stage of the viral life cycle at which the drug acts impacts the IC50 as well. As a general rule, the longer the drug-susceptible state exists (lower rates of decay *δ*_pre _or transition to the non-susceptible state *k*_HIV_), the lower the IC50, because there is more time for the drug to act and affect target cells. Finally, the faster the inhibited virus is depleted (higher *δ*_I_), the lower the IC50 because more susceptible cells are depleted before the drug dissociates and a transition to the non-susceptible state is possible. However, as the off-rate of the drug (*k*_*-I*_) decreases (i.e. there is less dissociation of the drug), the decay rate of inhibited virus, *δ*_I_, has less of an impact on the IC50.

### Cell-type specific kinetic properties of antiviral drugs may be related to viral life-cycle kinetics

While the IC50 of a drug is dependent on parameters related to the stage in the viral life cycle targeted by the drug (*δ*_pre_, *k*_HIV_, *δ*_I_), these parameters may also depend on the virus-producing cell type. Therefore, IC50 may vary with the type of host cell. To date, four primary cell types are known to support HIV replication to various degrees [18,19]: (i) activated CD4+ T cells, (ii) resting CD4+ T cells, (iii) macrophages, and (iv) monocytes. Activated CD4+ T cells and macrophages are the primary HIV-producing cell types and support all stages of the viral life cycle [20]. Kinetics of reverse transcription in activated CD4+ T cells are in general more rapid than in macrophages [18,19,21]. Our model would therefore predict that IC50s of HIV reverse transcriptase inhibitors would be lower in macrophages than in activated CD4+ T cells (see Section 1 of Additional File 1). Reverse-transcriptase-inhibitor IC50s measured in macrophages and in CD4+ T cells using comparable experimental methods are greatly limited in the literature. We have found that previously reported IC50s of five RT inhibitors in macrophages are on average 3 fold lower than IC50s measured using a similar and comparable single round infection assay in CD4+ T cells (see Section 1 of Additional File 1 and *Supplemental *Figure 1 of Additional File 2). Although this 3-fold difference is not statistically significant (p = 0.1422), the trend of the data is in the direction we predict; moreover, with only five drugs, the statistical power to resolve any existing difference is low. As analysis of currently available but sparse data is equivocal, future studies that quantify the kinetics of reverse transcription in macrophages and IC50 measurements of more drugs in macrophages will give better insight into our model predictions.

Two additional sources of viremia that may be of significance include pre-integration latently infected resting CD4+ T cells (PLIC) [22], which produce virions on T cell activation, and infected monocytes [19], which are believed to produce virus on cell differentiation into tissue macrophages. Although there are no published reports of IC50s for antiretroviral drugs using single-round infection assays in either PLIC or monocytes, we may use our model to predict how IC50 may be affected in these cell types. In both PLIC and monocytes, the kinetics of reverse transcription are slower than in productively infected CD4+ T cells and macrophages, respectively. Our model would therefore predict that the IC50 of reverse transcriptase inhibitors should be lower in PLIC and monocytes (see Section 2 of Additional File 1 and *Supplemental *Figure 2 in Additional File 3).

**Figure 2:**
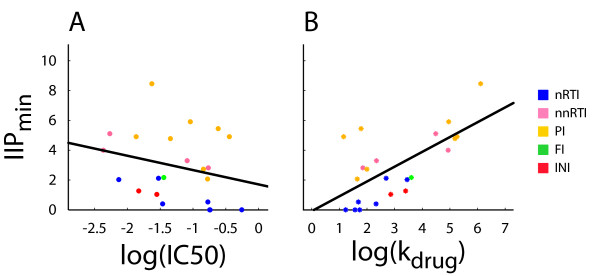
**Relationship of IIP_min _with IC50 and *k*_drug_**. log IIP_min _plotted against (A) log IC50 (represented by filled circles) or (B) log *k*_drug _(represented by stars; with *k*_drug _calculated as (*δ*_pre_+*k*_HIV_)/(IC50)*^m^*) for 25 different antiretroviral drugs (listed in Supplemental Table 1 of Additional File 1 and color-coded by drug class). Line of best fit through the data points in black. IIP and IC50 data from reference [5].

In the setting of HAART, HIV viremia decays with a triphasic decay. The first phase of decay represents declining virus production of productively infected, activated CD4+ T cells. The third phase of decay likely represents slowly decaying, latently infected resting CD4+ T cells. The identity of the virus-producing cells of the second phase, however, remains largely unknown. We have previously constrained the transition rate from post-reverse transcription to post-integration for the virus-producing cells of the second-phase decay to be quite low (with a half-life on the order of 1 week) [6-8,8]. Of all cell types that are infected by HIV, the kinetic constraints placed on these cells are most consistent with infected monocytes, where the slow progression from RT to integration may reflect the long time to differentiation into macrophages, followed by rapid integration (occurring in differentiated macrophages). In such a scenario, we would expect integrase inhibitors to have the same IC50 in the virus-producing cells of the second phase as in macrophages. However, it is possible that there exists another virus-producing cell type in which integration proceeds slowly and constantly to meet these kinetic constraints. In this scenario, our model predicts integrase inhibitors to have a lower IC50 in the virus-producing cells of the second phase than in macrophages. This predicted difference may help to identify the virus-producing cells of second-phase viremia.

### Drug-intrinsic determinants may be quantified using known kinetics of the viral life cycle

Thus far we have predicted that the experimentally observed IC50 of an antiretroviral drug (ARV) is determined by the kinetics of the viral life cycle but also by factors intrinsic to the ARV. In our model the parameter *k*_drug _is the amalgam of these ARV-intrinsic factors and is the rate at which a drug acts on its target, reflecting not only binding to the target but also intracellular transport, modification, or other direct interaction between the drug and the target cell. Our model may also be used to estimate the value of *k*_drug _for specific drugs. From equation 6, it is apparent that for drugs with low *k*_-I _compared to *δ*_I_, the IC50 will be predominantly determined by the kinetics of the stage in the viral life cycle at which the drug acts (i.e. *δ*_pre _and *k*_HIV_) and by *k*_drug_. In the case of productively infected CD4+ T cells and macrophages, experimental evidence of irreversibly or tightly binding antiretroviral drugs [23,24] suggests that *δ*_I _> >*k*_*-I*_. In this case, we can neglect *k*_-I _in (6), *δ*_I _cancels, and we obtain *k*_drug _≈ (*δ*_pre_+k_HIV_)/(IC_50_)^*m*^. The *in vitro *IC50s and *m*-values of twenty-seven ARVs have been previously reported [5]. Furthermore, the values of *δ*_pre _and *k*_HIV _have been reported or approximated for many of these drugs (see Section 3 of Additional File 1) and may therefore be used to solve for *k*_drug _(see *Supplemental *Table 1 in Additional File 1). We find that the values of *k*_drug _clearly show class-specific trends, with protease inhibitors having the largest *k*_drug _values in general. The instantaneous inhibitor potential (IIP) is an empirically derived measure of antiviral drug activity and reflects the log-fold decrease in viral replication by an ARV at a specified concentration [5]. IIP, which may be measured at the clinical minimum (IIP_min_), average (IIP_avg_), and maximum (IIP_max_) drug concentrations, reflects the best measurement of antiviral effect [5] in comparison to traditional pharmacodynamic properties, such as the IC50. When we reanalyzed the data from reference [5], we found poor correlation between the log(IC50) of antiretroviral drugs and their IIPs measured at their respective IIP_min _(spearman rank correlation, *ρ *= -0.323, P = 0.136) (Figure 2A), IIP_avg _(*ρ *= -0.365, P = 0.093), and IIP_max _(*ρ *= -0.356, P = 0.101) (see *Supplemental *Figures 3A and 3C in Additional File 4 for IIP_avg _and IIP_max_). In contrast, the calculated value of log(*k*_drug_) correlated very well with IIP_min _(*ρ *= 0.681, P = 0.0019) (Figure 2B), IIP_avg _(*ρ *= 0.729, P = 0.0008), and IIP_max _(*ρ *= 0.716, P = 0.0010), (see *Supplemental *Figures 3B and 3D in Additional File 4 for *IIP*_avg _and IIP_max_). At drug concentrations higher than IC50, IIP--calculated as -log(*f*_unaffected_)--is roughly equal to *m*log(*c*)-*m*log(IC150). It is therefore approximately a linear function of both *m *and log(IC50) if the respective other quantities are held constant (see Section 4 of Additional File 1 and *Supplemental *Figure 4 in Additional File 5). However, although the correlation between IIP and log(IC50) is not very strong (Figure 2A, *Supplemental *Figures 3A and 3C in Additional File 4), we find that IIP is very strongly correlated with the value of *m *(see Section 4 of Additional File 1 and *Supplemental *Figure 4 in Additional File 5). It is this strength of correlation between IIP and *m *that is the mathematical foundation for the correlation between IIP and log(*k*_drug_), which is also linear function of *m*. It is presently unclear why there is a strong correlation between IIP and *m *but not between IIP and IC50. One possible explanation is that the clinical concentrations at which IIP is measured tend to be chosen in proportion to the IC50. In this case, *c*/IC50 is going to be approximately constant and thus will not correlate with IIP. However, regardless of the reason why IIP correlates more strongly with *m *than with IC50, our results show that a parameter (*k*_drug_) designed *a priori *to purely reflect intrinsic drug properties (such as affinity for its target) correlates well with the experimentally measured suppression of viral replication.

**Figure 3:**
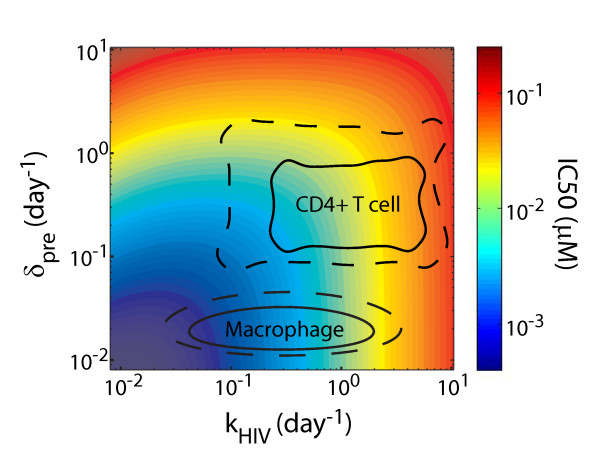
**IC50 color-coded as a function of δ_pre _and *k*_HIV_**. For a drug with *m *= 1 and *k*_drug _= 100, the parameter regimes generally occupied by the various stages of the HIV viral life cycle in activated CD4+ T cells and macrophages (for more details, see Section 5 of Additional File 1) are outlined with a solid line and parameter regime potentially available through pharmacologic intervention approximated with a dashed line based on cell-specific properties.

**Figure 4:**
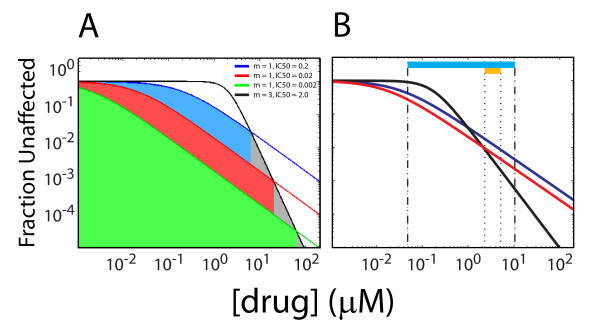
**Simulations that demonstrate how altering viral life cycle kinetics can improve drug efficacy**. Log-log concentration-response curves for (A) a hypothetical drug #1 and drug #2 where IC50_1 _> IC50_2 _but *m*_1 _= 1 and *m*_2 _= 3, showing how decreasing IC50 in drug #1 changes the concentration regime where each drug may have greater antiviral activity, and (B) with the example of how abacavir antiviral activity (blue concentration-response curve for IC50 = 0.0344 μM, red concentration-response curve for IC50 = 0.0172 μM) relative to nelfinavir (black concentration-response curve) may be improved by decreasing IC50. Light blue bar above indicates the clinical concentration range of abacavir and orange bar indicates clinical concentration range of nelfinavir.

Finally, because *k*_drug _is a function of not only binding of the antiretroviral drug to its enzymatic target but also of drug uptake, retention, and transport, experimental quantification of any of these factors will provide insight into the others, given their mathematical relationships in determining *k*_drug_. For example, when comparing different antiretroviral drugs from the same class, if their relative *k*_drug _values do not correlate with their relative rate of binding to the target HIV protein, then one may glean insight into the relative interaction of the drugs with the host cell (e.g. uptake, intracellular transport, modification, degradation, or expulsion).

### Antiretroviral activity may be improved through modifying the kinetics of the viral life cycle

Our results suggest that IC50 and therefore antiviral activity for a specific drug may be improved through modifying the kinetics of the viral life-cycle stage that is targeted by the drug. Such manipulation of viral life-cycle kinetics could occur through administration of an additional drug that may not be an efficient inhibitor of HIV *per se *but that modifies the viral kinetics in a beneficial manner. For example, decreasing the rate *k*_HIV _(e.g. reverse transcription) will decrease the IC50 of a reverse-transcriptase inhibitor by giving the inhibitor more time to act. However, manipulation of viral life-cycle kinetics is constrained by the physiologic extent to which these kinetics can be realistically altered in the different virus-producing cell-types (Figure 3).

In general, drug classes with greater concentration response sigmoidicity (i.e. a larger Hill coefficient *m*) should have better antiviral activity at drug concentrations much higher than their IC50. By contrast, drugs with lower *m *may have greater antiviral effect at drug concentrations far below their IC50s (Figure 4A), if the antiretroviral with higher *m *(drug #1 with *m*_1_) also has a higher IC50 than the antiretroviral drug with lower *m *(drug #2 with *m*_2_). This condition can arise for routinely used antiretroviral drugs such as the protease inhibitor nelfinavir (*m *= 1.81, IC50 = 0.1668 μm) and the nRTI abacavir (*m *= 0.95 and IC50 = 0.0344 μm). In these cases, the antiviral activity of the drug with higher *m *at concentrations exceeding its IC50 overcomes the antiviral activity of the drug with lower *m *at similar concentrations. The drug concentration at which the antiviral activity of the drug with higher *m *overcomes that of drug with lower *m *is the value of [*c*] at which the two *f*_unaffected _([*c*]) curves for the two drugs intersect. The value of *f*_unaffected _([*c*]) (for either drug) at this point is:

(7) $$f_{\text{unaffected}}^{*}=\frac{IC50_{1}^{\left[m_{1}^{2}+m_{1}\left(m_{2}-m_{1}\right)\right]∕\left(m_{2}-m_{1}\right)}}{IC50_{1}^{{}_{\left[m_{1}^{2}+m_{1}\left(m_{2}-m_{1}\right)\right]∕\left(m_{2}-m_{1}\right)}}+IC50_{2}^{m_{1}m_{2}∕\left(m_{2}-m_{1}\right)}}$$

which occurs at a drug concentration of $\left[c\right]={\left(IC50_{2}^{m_{2}}∕IC50_{1}^{m_{1}}\right)}^{1∕\left(m_{2}-m_{1}\right)}$.

If the activity of the antiretroviral drug with low *m *is increased through pharmacologic interventions aimed at the kinetics of the viral life cycle in order to decrease the IC50 (e.g. through increasing *k*_HIV _or *δ*_pre_) then the value of $f_{\text{unaffected}}^{*}$ decreases thereby decreasing the concentration range in which drug 2 is superior to drug 1 (Figure 4A). The effect of decreasing IC50 on lowering this *f*_unaffected _is best when $IC50_{2}^{m_{1}m_{2}∕\left(m_{2}-m_{1}\right)}>>IC50_{1}^{\left[m_{1}^{2}+m_{1}\left(m_{2}-m_{1}\right)\right]∕m_{1}m_{2}}$. However, even for the general case of a protease inhibitor like nelfinavir with *m *= 1.81 and IC50 = 0.1668 μM in comparison to an nRTI like abacavir with *m *= 0.95 and IC50 = 0.0344 μm, $f_{\text{unaffected}}^{*}$ can be reduced by 75% if the IC50 of abacavir is reduced by half (Figure 4B).

## Discussion

Continued progress in treatment of HIV infection depends not only on development of new antiretroviral drugs but also on complete understanding of how these drugs act. Such insight will allow for pharmacologic interventions that can further improve the action of known antiretroviral drugs. Recent experimental advances, such as the development of direct single round infection assays [4,25] that do not suffer from the unreliability of indirect or multiround assays [26,27] have provided a better understanding of the pharmacodynamic properties for available antiretroviral drugs. Moreover, class-specific pharmacodynamics properties that impact pharmacologic effect are now apparent as well. These properties reflected in pharmacodynamic parameters are obtained through the empirical fitting of models, such as the Michaelis-Menten or Median-Effect models of enzyme kinetics, to experimental data [10]. While these models can offer an empirical basis for understanding the pharmacodynamics and activity of antiretroviral therapy, they cannot provide mechanistic insight because HIV viral kinetics do not meet many of the important assumptions that underlie these models.

Here we present a simple and general mechanistic model of HIV interaction with antiretroviral drugs that takes into account the kinetics of the HIV viral life cycle. We have recently argued that the phase of the viral life cycle where a drug acts may have important implications for clinically observed viral dynamics [7]. Experiments *in vitro *have confirmed our argument [6]. Here, we demonstrate that the kinetics of the viral life cycle may also impact the pharmacodynamic properties of antiretroviral drugs. More broadly, we show that the IC50 of a drug is impacted by three categories of factors: (i) drug-intrinsic properties related to binding of drug to target, (ii) the kinetics of the stage of the viral life cycle at which the drug acts, and (iii) the kinetics of an infected cell that has been effectively inhibited. The theoretical framework provided by our model can be useful for gaining insight into either drug-intrinsic or -extrinsic factors when information about the other is available. Moreover, we have used the mathematical relationship between these factors that determine drug IC50 to quantify the drug-intrinsic properties (such as the strength and rate of interaction between an antiretroviral drug and its target) reflected by our model parameter *k*_drug _for 22 different antiretroviral drugs. Unlike experimentally measured IC50, we find that *k*_drug _correlates very well with experimentally measured antiviral activity for these drugs, as reflected by *IIP*. The mathematical underpinning of this correlation lays in the strong correlation we demonstrate between *IIP *and the parameter *m*. However, future experiments, which empirically fit the approximate value of *k*_drug _to dose-response data using the relationship f_unaffected _= 1/[1+*k*_drug_*c*^*m*^/(*δ*_pre_+*k*_HIV_)] (based on the relationship between *k*_drug _and IC50) will allow correlation between an independently obtained value of *k*_drug _and IIP.

The quantitative predictions of our model depend on accurate experimental measurements of model parameters for the different stages of the viral life cycle and in the different virus-producing cell types, which will also require experimental characterization of the drug-susceptible state. Furthermore, our model predictions may be confounded by other factors such as drug-specific interactions between other HIV machinery (e.g. excision machinery) or cellular machinery such as APOBEC3G as well as expression/activity level of MDR/P-glycoprotein pumps that may depend on drug concentration or be specific to the type of the host cell. Future experiments in this area will give greater insight into the magnitude of effect that such factors have on our model predictions.

The clinical efficacy of an antitretroviral drug is dependent on many factors such as the background regimen, adherence, resistance barrier, pharmacokinetics, and pharmacodynamics. While all of these factors represent avenues for improvement of currently existing drugs and development of new drugs, pharmacologic approaches to optimizing drug pharmacodynamics may allow for greater (1) flexibility in successful background regimens, (2) forgiveness in patient noncompliance, (3) protection from development of drug resistance in the cases of low resistance barriers and (4) antiviral activity given specific pharmacokinetic properties. Our results have important therapeutic implications, suggesting an approach to enhancing antiretroviral activity through pharmacologic interventions that may prolong the drug-susceptible state and opening the potential for drugs that may boost efficacy of antiretroviral drugs through modification of viral life-cycle kinetics. This prediction of our model may be tested experimentally through measurement of antiretroviral-drug IC50s in viruses carrying mutations specifically aimed at altering the drug-targeted stages of the viral life cycle (e.g. a mutation that slows the rate of reverse transcription). This effect may have already been clinically observed when hydroxyurea, which slows the process of reverse transcription by depleting the intracellular pools of dATP, was reported to enhance the activity of the reverse transcriptase inhibitor ddI, which specifically acts at the point of dATP insertion into the viral DNA [28]. If the functional effect of hydroxyurea is to slow the process of reverse transcription, our model predicts that the increased activity of ddI in the presence of hydroxyurea is due to a lower IC50. Moreover, our findings suggest that slowing the viral life cycle through pharmacologic interventions may serve as a means for at least partially overcoming drug resistance mutations that can increase the drug's IC50 [29].

Our results also have implications for the design of HAART regimens. Drugs acting within the same period of the viral life cycle but using different mechanisms of action may be used to combat resistance mutations. Resistance mutations often come at a fitness cost, for example through slowing HIV enzyme kinetics. Our model predicts that a mutation causing resistance to one drug may increase efficiency of the second drug. For example, consider resistance to one of two drugs acting at the same stage of the viral life cycle, e.g., a protease inhibitor and a maturation inhibitor. The mutation conferring resistance may slow the rate of progression for that stage of the viral life cycle (under the assumption that the wild-type virus is optimized to complete each stage of the viral life cycle as efficiently as possible). Our model predicts then that the antiviral activity of the other drug would increase due to a decreased IC50 resulting from slower kinetics. Such an effect has been observed for the combined use of protease and maturation inhibitors [30]. In the same vein, our results also suggest a mechanism that may be investigated for experimentally observed synergism or antagonism between antiretroviral drugs [31-33]. Furthermore, our model also predicts that IC50 and therefore antiviral activity may vary between different virus-producing cell types. After initiation of HAART, the dominant virus-producing cell changes through the three phases of viral decay (productively infected CD4+ T cells during the first phase; most likely monocyte/macrophages and latently infected CD4+ T cells during the second and third phases of decay, respectively). Our results suggest the optimization of a HAART regimen may ultimately require careful consideration of the prevalence of the different virus-producing cells both in the short term and the long term.

## Conclusions

Overall, our results suggest that consideration for the kinetics of the viral life cycle may offer substantial insight into the treatment of HIV. We find that *k*_drug _and *m *correlate well with antiviral activity, whereas IC50 does not. This finding suggests that IC50 may be confounded by kinetics of the viral life cycle. Moreover, our results predict not only underlying mechanisms that determine clinically observed pharmacologic properties of antiretroviral drugs but also suggest approaches for development of antiretrovirals including a novel approach for overcoming resistance through pharmacologic agents that affect the kinetics of the viral life cycle. Finally, our model and results will apply with minor changes to many other pathogens.

## Conflict of interest declaration

The authors declare that they have no competing interests.

## Authors' contributions

ARS conceived the study and performed simulations. ARS and COW developed the study, performed calculations and wrote the manuscript. All authors read and approved the final manuscript.

## Reviewers Comments

### Reviewer 1-Dr. Ruy Ribeiro

I appreciate the authors' thoughtful replies and I believe that the manuscript is substantially improved and affords better reading. I think that the main result of the manuscript namely "a simple and general mechanistic model of HIV interaction with antiretroviral drugs that that takes into account the kinetics of the HIV viral life cycle" is very interesting and a valuable contribution.

We would like to thank Dr. Ribeiro for the overall positive evaluation of our manuscript.

On the other hand, I still don't understand much of the discussion around IC50. Equation (6) that "links fundamental constants that describe how a drug acts to the experimentally observable IC50" is very nice. But the fact that IC50 by itself, without knowing the drug concentration, does not describe antiviral efficacy is not surprising. (In my previous comment I meant that IC50 "is the same as antiviral activity" at that concentration.) And the fact that IC50 is not a good way to compare activity across drug classes at different concentrations is again not surprising, and I am not sure it needs an explanation.

We agree that none of these facts are terribly surprising. Yet the way in which IC50 is commonly used in the literature often is at odds with these facts. Therefore, we think it is worth pointing them out.

I also don't agree with the explanation given for the statement "-log(f) is therefore a linear function of both m and IC50". A linear relationship implies that if the independent variable (say m, here) doubles and the dependent variable (say IIP) changes by a factor q; then when the independent variable quadruples the dependent variable changes by a factor 2q. This is not the case for the formula presented unless c/IC50 is approximately constant. There may still be some correlation (as in y and x in y = x^2), and you may be able to calculate a correlation coefficient. But the relationship is not linear for all values (although it may look linear for a certain range of the variables).

We are not sure whether Dr. Ribeiro's objection is that IIP = *m*log(*c*)-*m*log(IC50) is only approximately correct, in the limit of large *c*, or whether the objection is that the expression IIP = *m*log(*c*)-*m*log(IC50) does not imply that IIP is a linear function of *m *and IC50. We now write "approximately a linear function" to address the first possibility. In our mind, the expression IIP = *m*log(*c*)-*m*log(IC50)implies a linear relationship of IIP with *m *and log(IC50), since IIP changes linearly with either quantity if all other variables are held constant. To address the second possibility, we have added "if the respective other quantities are held constant."

### Reviewer 2-Dr. Ha Youn Lee

As the clarity of the manuscript is significantly improved, now I have three main comments.

First, we may understand the results of Figure 2A and Supplementary Figure 4 by assuming that *c*_min _(the clinical minimum concentration) is proportional to IC50. Since IIP_min _is approximately given by *m *log(*c*_min_/IC50), we observe a strong correlation between IIP_min _and *m *as in Supplementary Figure 4 and a poor correlation between IIPmin and IC50 as in Figure 2A, if *c*_min _and IC50 are happen to have a linear relationship within 25 antiretroviral drugs which are analyzed here.

We thank the reviewer for making this point and have added a few sentences at the appropriate location in the results.

Second, we might interpret the outcome of Figure 2B from the following relationship:

IIP_min _= log(*k*_drug_)-log(*δ*_pre _+ *k*_HIV_) + *m *log(*c*_min_).

According to the supplementary Table I, the values of *k*_drug _are greater than those of *δ*_pre _+ *k*_HIV _by several orders of magnitude. If *m *log(*c*_min_) is smaller than log(*k*_drug_) for most of the 25 drugs, we should be able to observe a linear relationship between log(*k*_drug_) and IIP_min _as log(*k*_drug_) is a leading order term.

It is true that IIP ≈ log(*k*_drug_)-log(*δ*_pre_+*k*_HIV_)+*m*log(*c*) if one makes the assumptions that 1) *c*/IC50 > > 1 and 2) *δ*_I _> >*k*_-I_. In the case of IIP_min_, the assumption that *c*_min_/IC150 > > 1 does not universally hold true. However the expression IIP_max _≈ log(*k*_drug_)-log(*δ*_pre_+*k*_HIV_)+*m*log(*c*_max_) would provide one mathematical explanation for why IIP_max _correlates with *c*_max_.

Third, I would like to encourage authors to revise both abstract and author summary by i) defining IIP and ii) adding more description about "the measure of intrinsic drug property".

i) Neither abstract nor author summary contain the term IIP. We prefer to leave this term out of both abstract and author summary because we believe that these pieces of text should contain as little jargon as possible.

ii) We have added the following sentence to the author summary: "We derive a measure of intrinsic drug properties that captures how well the drug is taken up, complexes with, and inhibits its target." We have left the abstract unchanged, since there already is an explanation of intrinsic drug properties in the abstract: "intrinsic drug properties (e.g. drug-target binding)"

### Reviewer 3-Dr. Alan Perelson

The revised manuscript is acceptable for publication. All of the concerns I raised in my previous review have been addressed.

We are glad to hear that Dr. Perelson considers our manuscript publication-ready.

### Reviewer 4-Dr. Christoph Adami

I am happy with the author's changes to the manuscript.

We are glad to hear that Dr. Adami considers our manuscript publication-ready.

## Supplementary Material

Additional file 1 **Supplemental Text**. Contains supplemental text and supplemental Table 1, all of which support results presented within the manuscript. Click here for file

Additional file 2 **Supplemental Figure 1: Experimentally reported IC50s for AZT, d4T, ddI, 3TC and TDF**. In CD4+ T cells [10] and macrophages [9]. Click here for file

Additional file 3 **Supplemental Figure 2: Log-log dose response curve for the reverse transcriptase inhibitor 3TC in different cell types**. For 3TC m = 1.15 and simulations are for infection in (A) activated CD4+ T cells (blue) (where *k*_HIV _= 8.32 day^-1^, *δ*_pre _= 0.3466 day^-1 ^and *δ*_I _= 0.3466 day^-1^) in contrast to PLIC (black) (where *k*_HIV _= 0.3466 day^-1^, *δ*_pre _= 0.231 day^-1 ^and *δ*_I _= 0.231 day^-1^); and (B) in macrophages (red) (where *k*_HIV _= 4.16 day^-1^, *δ*_pre _= 0.0495 day^-1 ^and *δ*_I _= 0.0495 day^-1^) in contrast to monocytes (black) (where *k*_HIV _= 0.231 day^-1^, *δ*_pre _= 0.0495 day^-1 ^and *δ*_I _= 0.0495 day^-1^). Click here for file

Additional file 4 **Supplemental Figure 3: Relationship of IIP_avg _and IIP_max _with IC50 and *k*_drug_**. log IIP_avg _plotted against (A) log IC50 (represented by filled circles) or (B) log *k*_drug _(represented by stars) for 25 different antiretroviral drugs (listed in Supplemental Table 1 of Additional File 1 and color-coded by drug class). log IIP_max _plotted against (C) log IC50 (represented by filled circles) or (D) log *k*_drug _(represented by stars). Line of best fit through data points in black. IIP and IC50 data from reference [10]. Click here for file

Additional file 5 **Supplemental Figure 4: IIPs plotted against *m***. IIP_min _(A), IIP_avg _(B) and IIP_max _(C) plotted against *m *for 25 different antiretroviral drugs listed in Supplemental Table 1 of Additional File 1. Line of best fit through data points in black. IIP and *m *data from reference [10]. Click here for file
