# Supplementary material for: Kinetics of the viral cycle influence pharmacodynamics of antiretroviral therapy
Source: Biol Direct. 2011 Sep 12;6:42. doi: 10.1186/1745-6150-6-42 (PMC3203257; doi:10.1186/1745-6150-6-42)
Supplement: Additional file 1 — Supplemental Text. Contains supplemental text and supplemental Table 1, all of which support results presented within the manuscript. [file 1745-6150-6-42-S1.DOC]

**Supplemental Materials**

**1. Pharmacodynamic properties of reverse transcriptase inhibitors in activated CD4+ T cells compared to macrophages**

Viral kinetics in activated CD4+ T cells are quite rapid, with total turnover time for a productively infected T cell on the order of one to two days [1, 2]. Therefore, the rate at which any one step of the viral life cycle proceeds to the next (e.g. reflected by $k_{\text{HIV}}$) will be even more rapid (with a half life on the order of hours) [3]. Similarly, activated CD4+ T cells are labile cells and so the decay rate of a drug-susceptible state (e.g. through death of the cell or decay of the viral complex) in the viral life cycle ($\delta_{\text{pre}}$) should be rapid as well. Therefore, because the IC50 is proportional to the sum $(\delta_{\text{pre}}+k_{\text{HIV}})^{1/m}$, reduction of either $\delta_{\text{pre}}$ or $k_{\text{HIV}}$ alone may not substantially reduce IC50, since the magnitudes of both $\delta_{\text{pre}}$ and $k_{\text{HIV}}$ in productively infected T cells are likely to be large.

By contrast, kinetics of the HIV life cycle in macrophages have not been well quantified. Previous work has demonstrated that macrophages are relatively resistant to the cytopathic effects of HIV [4, 5], with virus production on the order of 10 days [6-8], at least 10 times longer than in CD4+ T cells. We may glean from the published data that the drug-susceptible states in macrophages ($\delta_{\text{pre}}$) are likely longer-lived than in activated CD4+ T cells. The kinetics of reverse transcription, as they relate to the use of equation 6, are currently unknown for macrophages. However, the lower turnover rate of infected macrophages suggests that reverse transcription is slower in macrophages compared to CD4+ T cells and if so, our model would predict that the IC50 of reverse transcriptase inhibitors is lower in HIV infected macrophages compared to infected CD4+ T cells depending on the rates of reverse transcription in the two cell types. One recent study measured the IC50 of select nRTIs (AZT, d4T, ddI, 3TC and TDF) in macrophages using a single round drug assay [9]. We compared these IC50s to corresponding IC50s previously reported in activated CD4+ T cells [10]. Although these studies did differ in some ways such as indicator of infection—luciferase [9] vs. GFP [10]—and the use of human serum [10] vs. not [9], both studies [10] were performed under otherwise similar experimental conditions in primary cells using an NL4-3 backbone vector. We found that the IC50 of these drugs in macrophages is on average only 32% of that measured in CD4+ T cells (directional paired *t*-test on log-transformed IC50 values, *P* = 0.1422; see also Supplemental Figure 1 of *Additional File 2*). While this difference is not significant, the trend of the data is in the direction we predict; moreover, with only five drugs, the statistical power to resolve any existing difference is low. Future studies that quantify the kinetics of reverse transcription in macrophages and IC50 measurements of more drugs in macrophages will give better insight into our model predictions.

**2. Pharmacodynamic properties of reverse transcriptase inhibitors in pre-integration latently infected CD4 T+ cells and infected monocytes compared to productively infected CD4+ T cells and macrophages, respectively**

In both resting CD4+ T cells and monocytes, HIV replication is believed to be limited to early steps of the viral life cycle, proceeding only through reverse transcription [11-13]. Further progression through the viral life cycle in a resting CD4+ T cell depends on activation [11, 12] and in a monocyte depends on differentiation into a macrophage [13].

In comparison to activated CD4+ T cells, where reverse transcription occurs over the course of a few hours [3], the kinetics of reverse transcription are slower in PLIC, occurring over the course of 1 to 3 days [12, 14]. By contrast, the kinetics of integration and virion maturation in PLIC are expected to be the same as in activated CD4+ T cells, since these events occur only when the PLIC are activated. Therefore, while integrase inhibitors, protease inhibitors, and maturation inhibitors (currently in clinical trials) might be expected to have the same pharmacodynamics in PLIC as productively infected CD4+ T cells, we would expect reverse-transcriptase inhibitors to have a lower IC50 in PLIC than in productively infected cells, with an improved dose–response curve (Supplemental Figure 2A in *Additional File 3*). Because reverse transcription occurs on the order of a few hours in productively infected CD4+ T cells in contrast to the 1 to 3 days in PLIC, we would expect the IC50 of nucleoside reverse-transcriptase inhibitors (that have $m\approx1$ [10]) to be approximately between 4 to 20 fold lower and non-nucleoside reverse-transcriptase inhibitors (that have $m\approx2$ [10]) to be approximately 2 to 5 fold lower in PLIC compared to productively infected cells.

It is reported that monocytes infected by HIV may proceed in the viral life cycle through reverse transcription, after which point cellular differentiation into a macrophage is required for integration [11-13]. However, reverse transcription in monocytes occurs more slowly than in macrophages, in approximately 2.5 days [6]. We would therefore expect the IC50 of nucleoside reverse-transcriptase inhibitors (that have $m\approx1$ [10]) to be approximately between 4 to 20 fold lower and the IC50 of non-nucleoside reverse-transcriptase inhibitors (that have $m\approx2$ [10]) to be approximately 2 to 5 fold lower in monocytes compared to productively infected cells and macrophages (Supplemental Figure 2B in *Additional File 3*).

If infected monocytes must differentiate into macrophages for the viral life cycle to progress beyond reverse transcription, then we would expect integrase inhibitors, protease inhibitors, and maturation inhibitors to have the same pharmacodynamics in monocytes as in macrophages. However, it has also been suggested that HIV may possibly progress through the viral life cycle at an extremely slow rate in monocytes, with not only a slow rate of reverse transcription but also with a slow rate of integration and virus production [6]. In this case, we would expect the IC50 of integrase inhibitors, protease inhibitors, and maturation inhibitors to be lower in monocytes compared to macrophages. In fact, whether the IC50 of these agents is lower in monocytes compared to macrophages may help to elucidate whether monocytes can support viral replication at a lower rate or if they must differentiate into macrophages.

**3. Selection of model parameters $\delta_{\text{pre}}$ and $k_{\text{HIV}}$ for calculation of** $k_{\text{drug}}$

In order to calculate the approximate value of $k_{\text{drug}}$ for the various antiretroviral drugs CD4+ T cells, we required the values of $\delta_{\text{pre}}$ (the decay rate of the drug-susceptible state) and $k_{\text{HIV}}$ (the rate at which the drug-susceptible state progresses in the viral life cycle to become unsusceptible to the drug). The values of these parameters depend on the stage of the viral life cycle targeted by the various drugs. We approximate $\delta_{\text{pre}}$ to be 0.347 day^-1^ to reflect a two day half-life of the drug-susceptible state for all drugs except for fusion inhibitors. We choose this value of $\delta_{\text{pre}}$ to reflect the decay of rapidly activated CD4+ T cells [15]. We choose $\delta_{\text{pre}}$ to be 8.32 day^-1^ for fusion inhibitors to reflect the finding that free virions, exist for approximately 2 hrs on average before decaying [16, 17]. Free HIV virions have been reported to decay with a half-life as low as 45 minutes [18]. However, whether a 45 minute half-life is used or a 2 hr half-life is used, our main results are not affected.

We next determined the values of $k_{\text{HIV}}$ for each drug class. The rate of reverse transcription in activated CD4+ T cells has been reported to be on the order of 2 hrs for an HIV genome of roughly 10^4^ nucleotides in activated CD4+ T cells [19, 20]. The nRTI drug class acts by becoming directly incorporated in the provirus and we therefore set $k_{\text{HIV}}$ = 8.32 day^-1^ to reflect the average time it takes for this process. The nnRTI drug class also inhibits reverse transcription but indirectly by binding to the reverse transcriptase enzyme. Because the reverse transcriptase may be susceptible to this drug after viral entry but before reverse transcription, we set $k_{\text{HIV}}$ = 4.16 day^-1^ to reflect not only the average time for reverse transcription but also two hours from entry to completion of reverse transcription. Protease inhibitors may impact a virion from the time that it forms and matures before budding, through the free virion phase to the final steps of maturation after viral entry into the target cell. We choose $k_{\text{HIV}}$ = 1.39 day^-1^ to reflect the, at most, 12 hrs it takes for these processes [2, 20]. The fusion inhibitor T20 acts by binding to gp41 during the entry process. We therefore choose $k_{\text{HIV}}$ = 8.32 day^-1^ to reflect the approximately 2 hrs it takes on average for the entry process [21]. Finally, integrase inhibitors raltegravir and elvitegravir act to inhibit the HIV integrase enzyme during the process of integration. We therefore set $k_{\text{HIV}}$ = 24 day^-1^ [22] to reflect the rate of integration as previously reported based on cell free experiments.

**4. The dominant factor in determining IIP is *m***

The instantaneuous inhibitory potential (IIP) is previously [10] defined as $-\log(f_{\text{unaffected}})$, which is equal to $\log[1+(c/\text{IC50})^{m}]$. For drug concentrations (*c*) that are larger than IC50, $\text{IIP}\approx m\log(c/\text{IC50})$. Previously [10], IIP has been calculated at the *in vivo* minimum, average, and maximum drug concentrations (*IIP*_min_, *IIP*_avg_, and *IIP*_max_). With the knowledge that these drug concentrations as well as respective IC50s vary for the different antiviral drugs, we correlated the value of *m* with the *IIP* at the *in vivo* minimum, average, and maximum drug concentrations. We found that *m* correlates very strongly with *IIP*_min_ ($\rho$ = 0.888, P<10^-6^), *IIP*_avg_ ($\rho$ = 0.911, P<10^-6^), and *IIP*_max_ ($\rho$ = 0.898, P<10^-6^) (Supplemental Figure 4A, B and C, respectively in *Additional File 5*).

**5. Determination of $\delta_{\text{pre}}$ and $k_{\text{HIV}}$ ranges in Figure 3**

The parameter ranges for $\delta_{\text{pre}}$ and $k_{\text{HIV}}$ generally occupied by the various stages of the viral life cycle in CD4+ T cells and macrophages are approximated based on experimental data using a solid line. For the ranges of $\delta_{\text{pre}}$ and $k_{\text{HIV}}$ occupied by CD4+ T cells, see section 3 of *Supplemental Materials* above. For the range of $\delta_{\text{pre}}$ occupied by macrophages, we considered the experimental evidence that macrophages are highly resistant to HIV infection, with a decay rate on the order of 0.0145 day^-1^ [16]. Unfortunately, $k_{\text{HIV}}$ is not well characterized for most stages of the viral life cycle in macrophages. However, we assume that the rate $k_{\text{HIV}}$ cannot be slower than the decay rate of macrophages (~ 0.0145 day^-1^) and use this as a lower bounds. We also consider that some processes such as virus binding and entry (targeted by fusion and entry inhibitors) likely take place at approximately the same rate as CD4+ T cells (i.e. there are no obvious physiologic restrictions). We therefore set the upper bounds of $k_{\text{HIV}}$ to be on the order of 10^1^.

Unfortunately, in neither CD4+ T cells nor macrophages is the parameter $k_{\text{HIV}}$ fully characterized for all drugs. Continued experimental quantitation of this rate for the various stages of the viral life cycle will allow for more accurate representation.

Dotted lines in Figure 5 represent approximations for ranges of $\delta_{\text{pre}}$ and $k_{\text{HIV}}$ In CD4+ T cells and macrophages that may be obtained through pharmacologic intervention. These approximations are made with cell-specific properties in mind, such as the cytopathic effect of HIV replication in CD4+ T cells, which will likely limit the how slow the rate $k_{\text{HIV}}$ may become.

**Supplemental Table 1. Pharmacodynamic** parameters of antiretroviral drugs

| **Class^1^** | **Name^2^** | **IC50^3^** (μM) | **m-value^3^** | ***δ*_pre_** (day^-1^) | ***k*_HIV_** (day^-1^) | $k_{\text{drug}}$ |
| --- | --- | --- | --- | --- | --- | --- |
| nRTI | 3TC | 0.0298 | 1.15 | 0.3466 | 8.32 | 492.48 |
|  | ABC | 0.0344 | 0.95 | 0.3466 | 8.32 | 212.82 |
|  | AZT | 0.1823 | 0.85 | 0.3466 | 8.32 | 36.82 |
|  | d4T | 0.5524 | 1.13 | 0.3466 | 8.32 | 16.94 |
|  | ddI | 0.1794 | 1.07 | 0.3466 | 8.32 | 54.47 |
|  | FTC | 0.0074 | 1.18 | 0.3466 | 8.32 | 2831.67 |
|  | TDF | 0.1684 | 0.97 | 0.3466 | 8.32 | 48.77 |
| nnRTI | EFV | 0.0054 | 1.69 | 0.3466 | 4.16 | 30619.17 |
|  | NVP | 0.0814 | 1.55 | 0.3466 | 4.16 | 219.92 |
|  | DLV | 0.1713 | 1.56 | 0.3466 | 4.16 | 70.64 |
|  | ETR (TMC125) | 0.0043 | 1.81 | 0.3466 | 4.16 | 86528.85 |
|  | TMC278 | 0.0039 | 1.92 | 0.3466 | 4.16 | 190061.77 |
| PI | APV | 0.1442 | 2.09 | 0.3466 | 1.39 | 99.20 |
|  | ATV | 0.0136 | 2.69 | 0.3466 | 1.39 | 181779.88 |
|  | DRV | 0.0236 | 3.61 | 0.3466 | 1.39 | 1295845.24 |
|  | IDV | 0.0909 | 4.53 | 0.3466 | 1.39 | 90463.02 |
|  | LPV | 0.0358 | 2.05 | 0.3466 | 1.39 | 1597.00 |
|  | NFV | 0.1668 | 1.81 | 0.3466 | 1.39 | 44.32 |
|  | SQV | 0.0453 | 3.68 | 0.3466 | 1.39 | 152870.64 |
|  | TPV | 0.242 | 2.51 | 0.3466 | 1.39 | 61.01 |
| FI | T20 | 0.0359 | 1.65 | 8.32 | 8.32 | 4028.38 |
|  | T1249 | 0.0112 | 1.97 | 8.32 | 8.32 | 57949.17 |
| INI | L870812 | 0.2282 | 0.99 | 0.3466 | 24.0 | 105.12 |
|  | RAL | 0.0149 | 1.10 | 0.3466 | 24.0 | 2488.47 |
|  | EVG (GS9137) | 0.0281 | 0.95 | 0.3466 | 24.0 | 724.71 |
|  | L240 | 0.0152 | 1.24 | 0.3466 | 24.0 | 4374.74 |
|  | L525 | 0.0861 | 1.00 | 0.3466 | 24.0 | 282.77 |

^1^Drug class abbreviations: nRTI (nucleoside-analogue reverse transcriptase inhibitor), nnRTI (non-nucleoside reverse transcriptase inhibitor), PI (protease inhibitor), FI (fusion inhibitors), and INI (integrase inhibitors).

^2^Drug name abbreviations: 3TC (lamivudine), ABC (abacavir), AZT (zidovudine), d4T (stavudine), ddI (didanosine), FTC (emtricitabine), TDF (tenofovir), EFV (efavirenz), NVP (nevirapine), DLV (delavirdine), ETR (etravirine), APV (amprenavir), ATV (atazanavir), DRV (darunavir), IDV (indinavir), LPV (lopinavir), NFV (nelfinavir), SQV (saquinavir), T20 (enfuvirtide), RAL (raltegravir) and EVG (elvitegravir).

^3^From reference [10]

References

1.     Markowitz M, Louie M, Hurley A, Sun E, Di Mascio M, et al. (2003) A novel antiviral intervention results in more accurate assessment of human immunodeficiency virus type 1 replication dynamics and T-cell decay in vivo. J Virol 77(8): 5037-5038.

2.     Rouzine IM, Sergeev RA, Glushtsov AI. (2006) Two types of cytotoxic lymphocyte regulation explain kinetics of immune response to human immunodeficiency virus. Proc Natl Acad Sci U S A 103(3): 666-671.

3.     Vatakis DN, Nixon CC, Bristol G, Zack JA. (2009) Differentially stimulated CD4+ T cells display altered human immunodeficiency virus infection kinetics: Implications for the efficacy of antiviral agents. J Virol 83(7): 3374-3378.

4.     Stevenson M. (2003) HIV-1 pathogenesis. Nat Med 9(7): 853-860.

5.     Marchant D, Neil SJ, McKnight A. (2006) Human immunodeficiency virus types 1 and 2 have different replication kinetics in human primary macrophage culture. J Gen Virol 87(Pt 2): 411-418.

6.     Arfi V, Riviere L, Jarrosson-Wuilleme L, Goujon C, Rigal D, et al. (2008) Characterization of the early steps of infection of primary blood monocytes by human immunodeficiency virus type 1. J Virol 82(13): 6557-6565.

7.     Gousset K, Ablan SD, Coren LV, Ono A, Soheilian F, et al. (2008) Real-time visualization of HIV-1 GAG trafficking in infected macrophages. PLoS Pathog 4(3): e1000015.

8.     Brown A, Zhang H, Lopez P, Pardo CA, Gartner S. (2006) In vitro modeling of the HIV-macrophage reservoir. J Leukoc Biol 80(5): 1127-1135.

9.     Perez-Bercoff D, Wurtzer S, Compain S, Benech H, Clavel F. (2007) Human immunodeficiency virus type 1: Resistance to nucleoside analogues and replicative capacity in primary human macrophages. J Virol 81(9): 4540-4550.

10.     Shen L, Peterson S, Sedaghat AR, McMahon MA, Callender M, et al. (2008) Dose-response curve slope sets class-specific limits on inhibitory potential of anti-HIV drugs. Nat Med 14(7): 762-766.

11.     Pierson TC, Zhou Y, Kieffer TL, Ruff CT, Buck C, et al. (2002) Molecular characterization of preintegration latency in human immunodeficiency virus type 1 infection. J Virol 76(17): 8518-8531.

12.     Zhou Y, Zhang H, Siliciano JD, Siliciano RF. (2005) Kinetics of human immunodeficiency virus type 1 decay following entry into resting CD4+ T cells. J Virol 79(4): 2199-2210.

13.     Triques K, Stevenson M. (2004) Characterization of restrictions to human immunodeficiency virus type 1 infection of monocytes. J Virol 78(10): 5523-5527.

14.     Koelsch KK, Liu L, Haubrich R, May S, Havlir D, et al. (2008) Dynamics of total, linear nonintegrated, and integrated HIV-1 DNA in vivo and in vitro. J Infect Dis 197(3): 411-419.

15.     Kovacs JA, Lempicki RA, Sidorov IA, Adelsberger JW, Herpin B, et al. (2001) Identification of dynamically distinct subpopulations of T lymphocytes that are differentially affected by HIV. J Exp Med 194(12): 1731-1741.

16.     Perelson AS, Neumann AU, Markowitz M, Leonard JM, Ho DD. (1996) HIV-1 dynamics in vivo: Virion clearance rate, infected cell life-span, and viral generation time. Science 271(5255): 1582-1586.

17.     Mittler JE, Markowitz M, Ho DD, Perelson AS. (1999) Improved estimates for HIV-1 clearance rate and intracellular delay. AIDS 13(11): 1415-1417.

18.     Ramratnam B, Bonhoeffer S, Binley J, Hurley A, Zhang L, et al. (1999) Rapid production and clearance of HIV-1 and hepatitis C virus assessed by large volume plasma apheresis. Lancet 354(9192): 1782-1785.

19.     Abbotts J, Jaju M, Wilson SH. (1991) Thermodynamics of A:G mismatch poly(dG) synthesis by human immunodeficiency virus 1 reverse transcriptase. J Biol Chem 266(6): 3937-3943.

20.     Reddy B, Yin J. (1999) Quantitative intracellular kinetics of HIV type 1. AIDS Res Hum Retroviruses 15(3): 273-283.

21.     Wang J, Kondo N, Long Y, Iwamoto A, Matsuda Z. (2009) Monitoring of HIV-1 envelope-mediated membrane fusion using modified split green fluorescent proteins. J Virol Methods 161(2): 216-222.

22.     Andreadis ST, Palsson BO. (1996) Kinetics of retrovirus mediated gene transfer: The importance of intracellular half-life of retroviruses. J Theor Biol 182(1): 1-20.
