# Supplementary figures and images for: Kinetics of the viral cycle influence pharmacodynamics of antiretroviral therapy
Source: Biol Direct. 2011 Sep 12;6:42. doi: 10.1186/1745-6150-6-42 (PMC3203257; doi:10.1186/1745-6150-6-42)

# Supplemental Figure 1

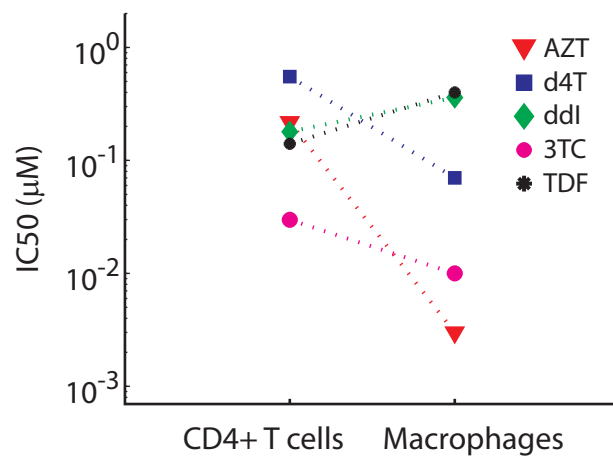

Supplement: Additional file 2 — Supplemental Figure 1: Experimentally reported IC50s for AZT, d4T, ddI, 3TC and TDF. In CD4+ T cells [10] and macrophages [9]. [file 1745-6150-6-42-S2.PDF]

## Supplemental Figure 2

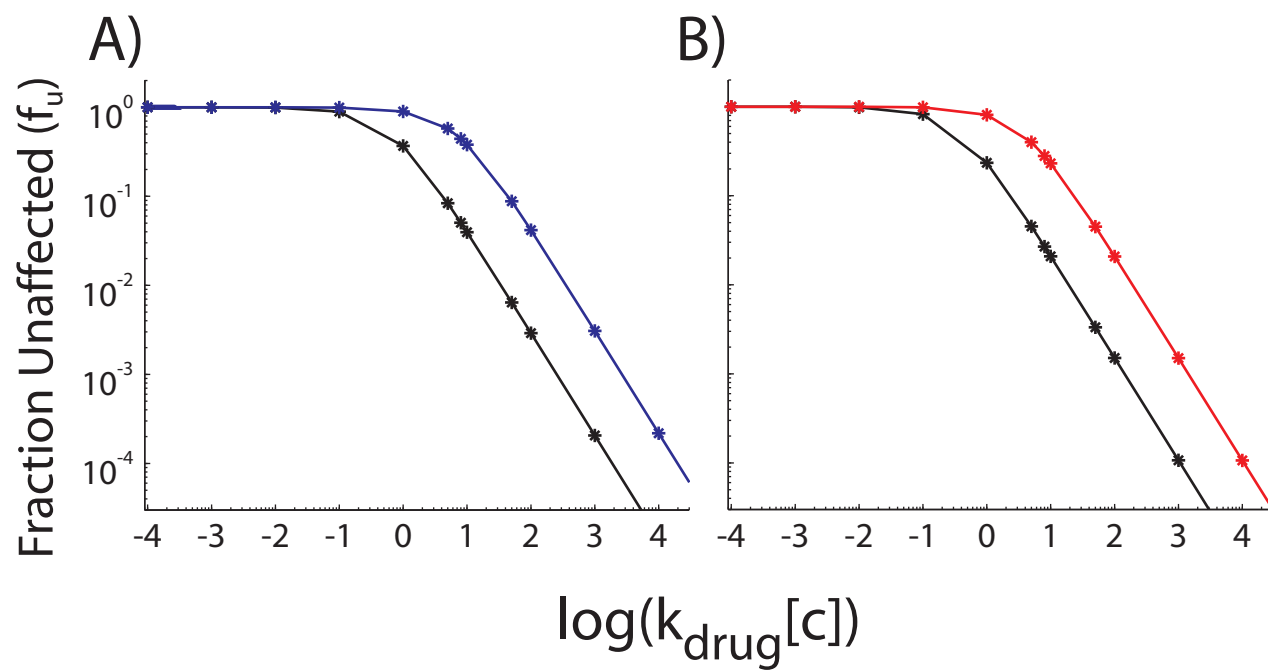

Supplement: Additional file 3 — Supplemental Figure 2: Log-log dose response curve for the reverse transcriptase inhibitor 3TC in different cell types. For 3TC m = 1.15 and simulations are for infection in (A) activated CD4+ T cells (blue) (where kHIV = 8.32 day-1, δpre = 0.3466 day-1 and δI = 0.3466 day-1) in contrast to PLIC (black) (where kHIV = 0.3466 day-1, δpre = 0.231 day-1 and δI = 0.231 day-1); and (B) in macrophages (red) (where kHIV = 4.16 day-1, δpre = 0.0495 day-1 and δI = 0.0495 day-1) in contrast to monocytes (black) (where kHIV = 0.231 day-1, δpre = 0.0495 day-1 and δI = 0.0495 day-1). [file 1745-6150-6-42-S3.PDF]

# Supplemental Figure 3

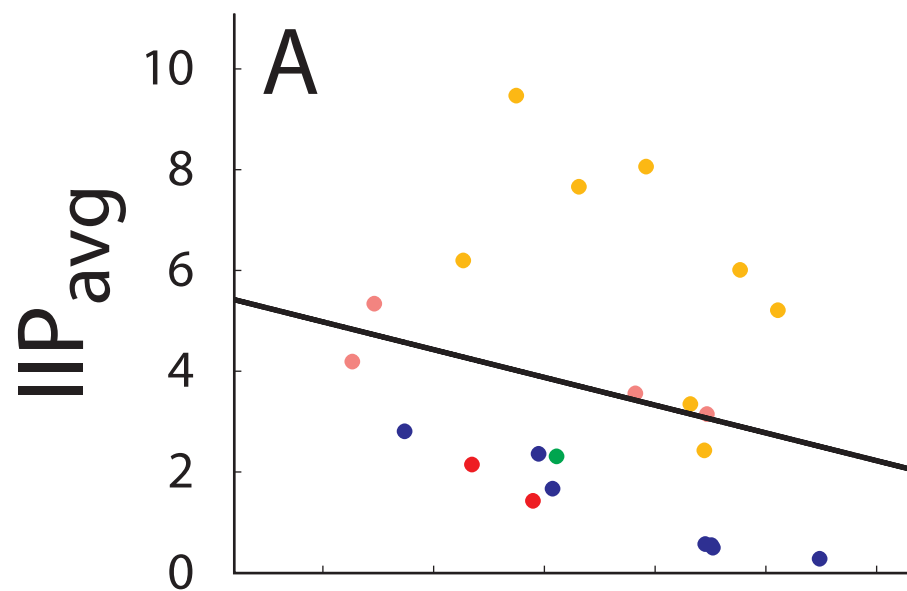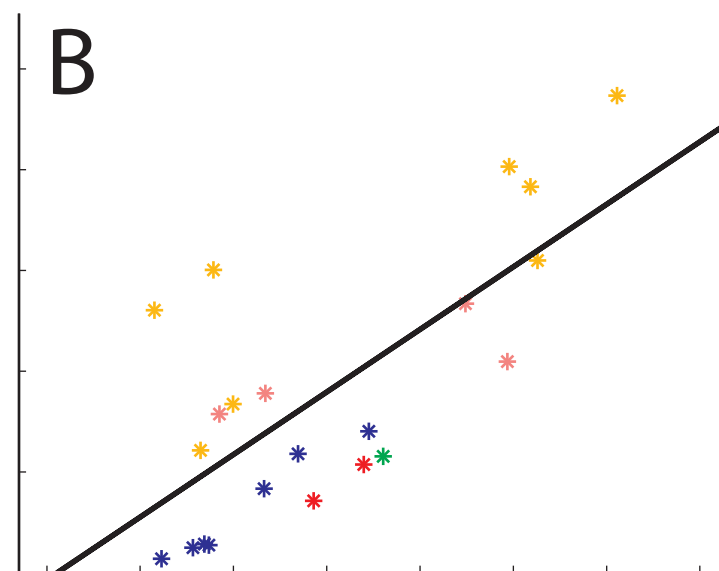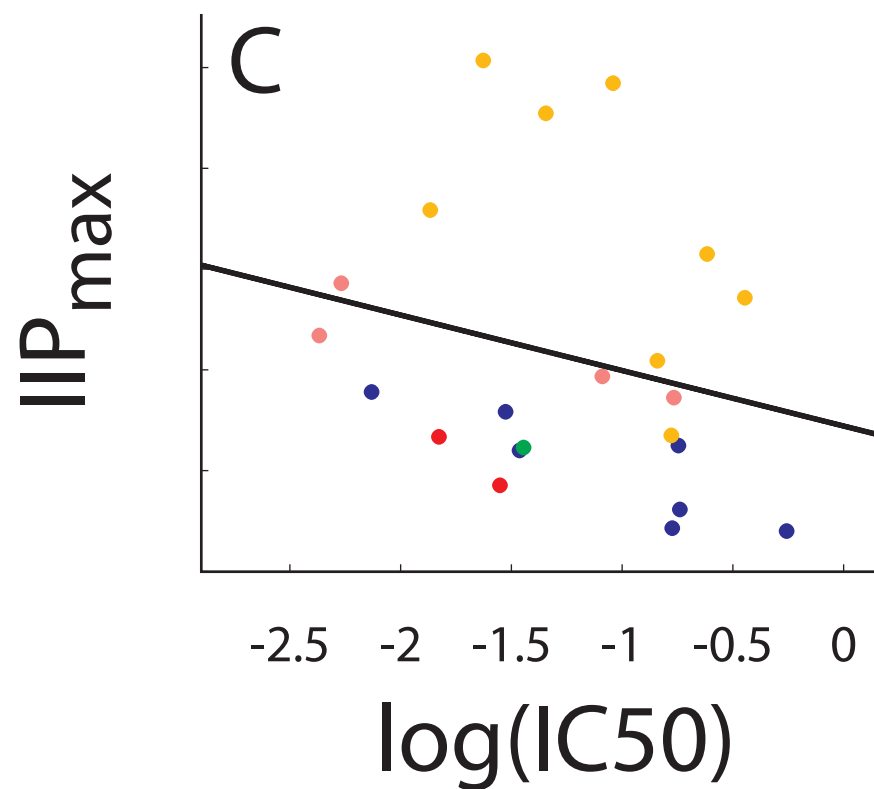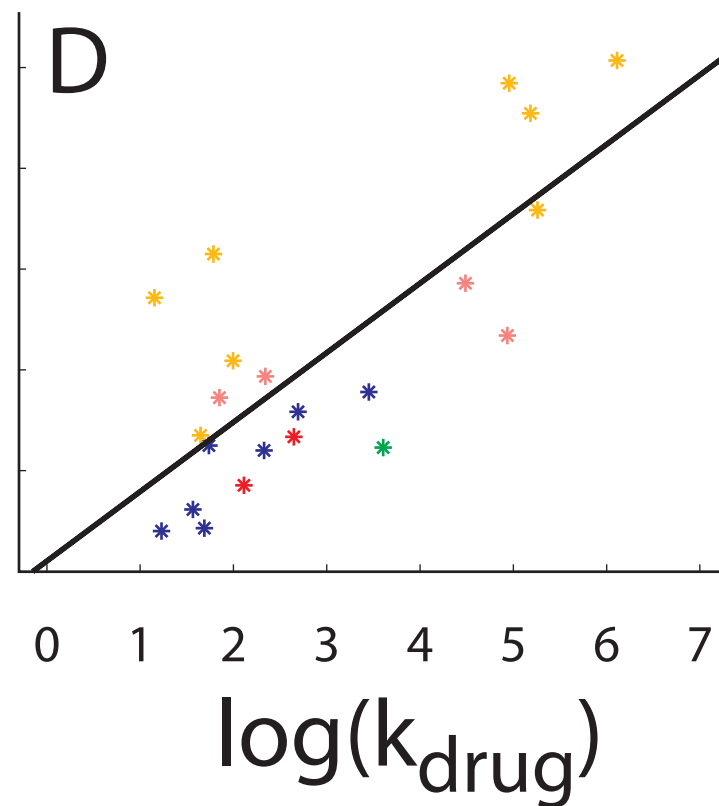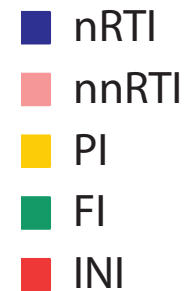

Supplement: Additional file 4 — Supplemental Figure 3: Relationship of IIPavg and IIPmax with IC50 and kdrug. log IIPavg plotted against (A) log IC50 (represented by filled circles) or (B) log kdrug (represented by stars) for 25 different antiretroviral drugs (listed in Supplemental Table 1 of Additional File 1 and color-coded by drug class). log IIPmax plotted against (C) log IC50 (represented by filled circles) or (D) log kdrug (represented by stars). Line of best fit through data points in black. IIP and IC50 data from reference [10]. [file 1745-6150-6-42-S4.PDF]

# Supplemental Figure 4

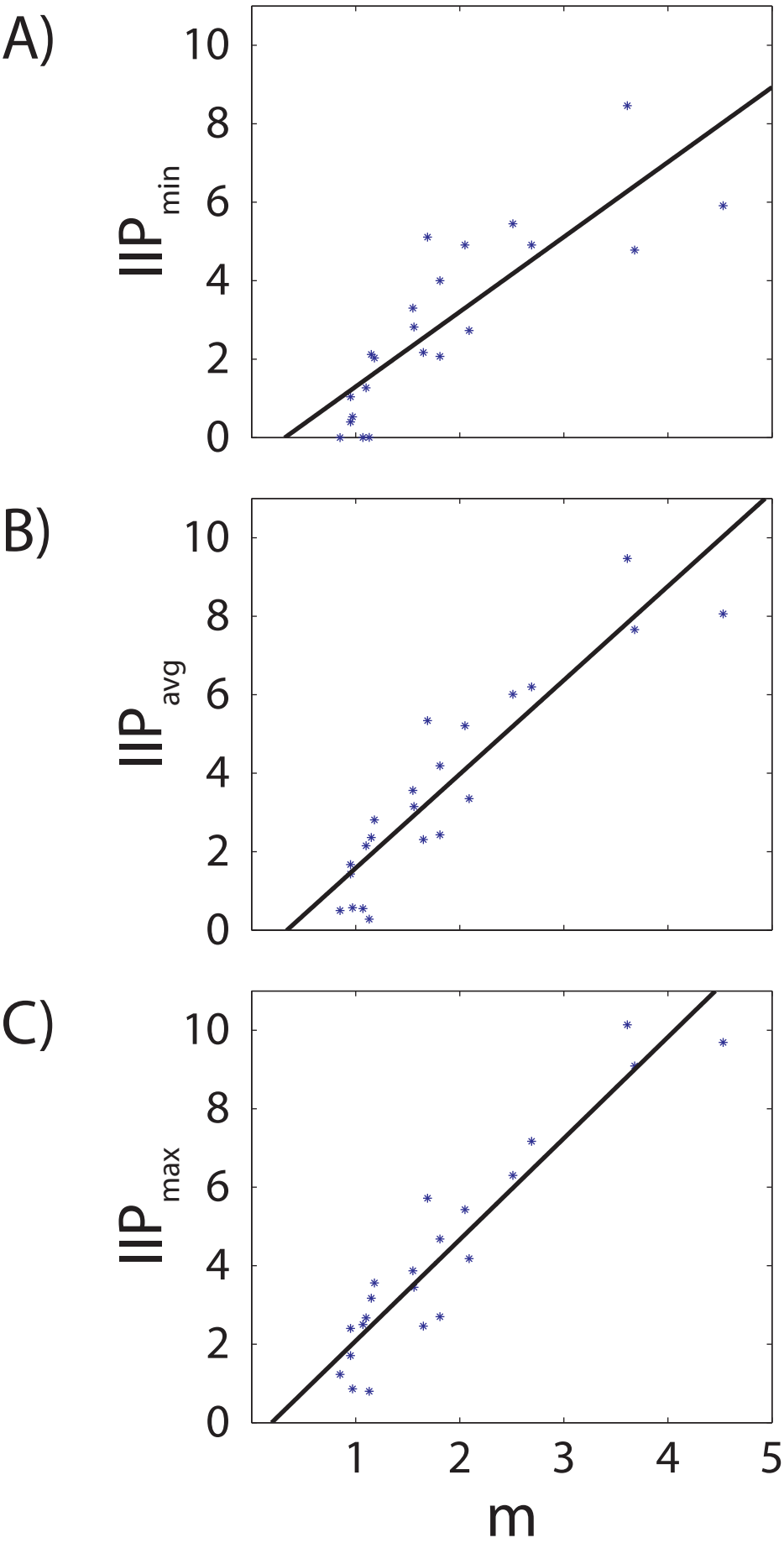

Supplement: Additional file 5 — Supplemental Figure 4: IIPs plotted against m. IIPmin (A), IIPavg (B) and IIPmax (C) plotted against m for 25 different antiretroviral drugs listed in Supplemental Table 1 of Additional File 1. Line of best fit through data points in black. IIP and m data from reference [10]. [file 1745-6150-6-42-S5.PDF]
